# Genomic Regions Associated with Variation in Pigmentation Loss in Saddle Tan Beagles

**DOI:** 10.3390/genes12020316

**Published:** 2021-02-23

**Authors:** Mia E. Nord, Per Jensen

**Affiliations:** IFM Biology, Linköping University, 58186 Linköping, Sweden; mia.persson88@gmail.com

**Keywords:** dog, fur color, MITF, domestication

## Abstract

Loss of pigmentation is a hallmark of domestication, and dogs offer a unique model for understanding the genetics of fur coloration. The aim of this study was to use dense genetic mapping to map loci underlying variations in color and whiteness in a population of laboratory beagles. A total of 190 beagles with well-defined pedigrees were phenotyped for the amount of white color in six different body parts, including the saddle. All individuals were genotyped on 85,172 informative and valid SNP-markers and the genome-wide associations for the amount of white in each body part were determined. There was a large variation in the amount of white on different parts of the body, and the whiteness was highly correlated within individuals, except for saddle color which was only moderately correlated with overall whiteness. The GWAS showed significant associations with two loci, one on chromosome 5, containing the *MC1R* gene, and one on chromosome 20, containing the *MITF* gene. Our results suggest that the variation in loss of pigmentation is largely a function of regulatory variation related to these genes.

## 1. Introduction

Loss of pigmentation is one of the defining phenotypic traits differing domesticated animals from their wild ancestors, and central to what Darwin [1] referred to as the domestication syndrome. The famous Siberian farm fox experiment, where foxes were selected on one behavioral trait only, i.e., friendliness towards humans, found that loss of pigment and piebald white spotting started to appear in the selected population after only 8–10 generations [2]. Hence, increased understanding of the genetics underlying variation in pigmentation of domestic animals may pave the way for closer insights into the mechanisms of domestication. For example, in chickens, a mutation in the gene *PMEL17* causes a total loss of black pigmentation and also pleiotropically affects domestication-related behavior [3].

Due to the unique domestication and population history of the dog, it now serves as an ideal subject to investigate genetic associations for behavioral as well as morphological traits [4]. Through the process of domestication and selection by humans, the domestic dog (*Canis familiaris*) has become an impressively diverse species with its almost 400 different breeds with different combinations of fur coloration, color patterns, length, and textures. Some breeds are, more or less, fixed for a fur coloration phenotype e.g., the golden retriever, while other breeds come in a wide variety of colors and patterns such as the boxer. In wolves, the ancestors of all dogs, there is a limited color variation, where most individuals are camouflaged in brown and grey, but alleles for melanism as well as light hair color appear to have been present in the wolf population more than 10,000 years ago [5]. Some of the present-day variation in whiteness could therefore be due to selection of standing variation in the ancestral wolf population, but new mutations in pigmentation-related genes have probably been selected as well.

Coat coloration and pattern in mammals is caused by the production of just two types of pigments: black-brown eumelanin and red-yellow pheomelanin [6]. These pigments are produced by melanocytes in organelles called melanosomes that are then transferred out to hair and skin cells. Precursor cells of melanocytes migrate from the neural crest to hair follicles and epidermis during embryogenesis. White spotting and loss of pigmentation is usually due to an absence of melanocytes caused by e.g., failure in differentiation during development or migration. Although many details of the genetic mechanisms underlying coat color are still insufficiently known, some aspects are more well-described. For example, Melanocortin 1 receptor (*MC1R*) controls which type of pigment is synthesised by the melanocytes. High *MC1R* signaling leads to increased eumelanin synthesis. In turn, Agouti signaling protein (*ASIP*) can inhibit *MC1R*, causing an increase in pheomelanin synthesis. Additionally, *CBD103* can bind to MC1R and block the binding site of *ASIP* and in that way increase the synthesis of eumelanin. Other genes are involved as well, for example, *TYRP1* that is associated with brown color, and *MLPH*, involved in diluted colors [7]. A recent review summarises the state of art of the complex genetic underpinnings of dog fur color genetics [8].

Based on inheritance, Little [9] defined the genetic nomenclature of dog coat color alleles still used today (summarised by Kaelin and Barsh [6]). For example, Little described the S-locus (Spotting), subsequently mapped to the *MITF*-gene, E-locus (Extension), later mapped to the *MC1R*-gene, the A-locus (Agouti), later mapped to the *ASIP*-gene and the *RALY*-gene that Little originally believed was an allele of the A-locus. Today, we are able to use powerful analyses such as genome-wide association studies (GWAS) to further disentangle the different loci proposed by Little and identify molecular pathways associated with fur coloration. For example, recent studies have identified regulatory modules for ventral as well as hair cycle *ASIP* expression, including their evolutionary origins, and found no association with *RALY* [10].

Beagles are considered fixed for the saddle tan and piebald fur coloration genotype [11,12,13]. This means that all individuals have at least some white markings on the paws, tip of the tail, and on the ventral side of the body including the neck caused by the *s^p^/s^p^*- genotype of the *MITF*-gene. Additionally, all individuals that are able to express eumelanin at the *MC1R*-gene will have a saddle on top of a tan background. The saddle is the black fur covering the dorsal area of the body, in beagles, it typically includes the neck and reaches all the way to the base of the tail. According to the data presented by Dreger and Schmutz [14] and Dreger, Hooser [12], beagles genotype *a^t^/a^t^* at the *ASIP* gene which is required for the saddle tan and black-and-tan phenotypes.

In the beagle laboratory population studied by us, there is a vast range of variation in the amount of white spotting and the amount of black coloration of the saddle. This suggests that other modifier loci than those previously identified are involved in affecting the expression of fur coloration, and in consequence affecting the degree of loss of pigmentation. The aim of this study was therefore to identify genomic region associated with variation in loss of pigmentation on different body parts in a population of laboratory beagles.

## 2. Materials and Methods

### 2.1. Animals

The 190 laboratory beagles phenotyped and genotyped in this study were originally used for another GWAS on human-directed social behaviors [15]. All dogs belonged to the same research population and were on average 1.8 years old (min 0.8 years, max 6.2 years) at the time of phenotyping. The population was intentionally outbred; however, some degree of inbreeding has to be accounted for in the analysis. For a more detailed description on housing, handling, and behavioural phenotyping see Persson and Roth [16].

### 2.2. Phenotyping and Genotyping

Screen shots of all dogs were collected from video recordings. At least three pictures (left side, right side, and frontal view of the face) from each individual were saved in order to phenotype their white patches of the face, neck, legs, body, and tail as well as the black saddle. Each individual was then subjectively scored on a scale from 1 to 3 based upon the coverage of their white spotting and the black saddle (Table 1). The reliability of the phenotype scoring was assessed by an independent observer recoding 10% of the individuals (N = 19) and then calculating the Spearman correlation coefficients to the two independent classifiers. Except for three individuals, all dogs were tri-colored black and tan with white spotting. However, three individuals were only expressing tan and white spotting and were therefore excluded from the saddle analysis as they may still carry the genetic variants for the saddle without being able to express the phenotype.

DNA was obtained from either blood or buccal cells and subsequently genotyped using Illumina’s Infinium assay and the CanineHD BeadChip at the SNP&SEQ Technology Platform, Uppsala University in Sweden, based on the CanFam3 assembly. To confirm sample and marker quality, SNP&SEQ also performed initial quality control using the software GenomeStudio 2011.1 from Illumina Inc. [17]. No samples had to be excluded since all had a call rate > 98% and therefore passed the 90% threshold.

Additional quality control was performed on 172,115 markers using the PLINK 1.07 software (http://pngu.mgh.harvard.edu/~purcell/plink/ (accessed on 20 January 2021)) [18]. Markers failing the criteria of minor allele frequency (MAF) > 5%, Hardy–Weinberg *p* > 0.05 and genotyping rate >90% were removed. Finally, 85,172 markers passed the criteria and were used in the GWAS. To visualize population structure, multidimensional scaling (MDS) analysis was performed in PLINK and the first and second dimensions were plotted.

### 2.3. GWAS

A minor level of population stratification (genomic inflation factor λ = 1.0–1.4) is present within the studied population [15]. To adjust for relatedness as well as population stratification the GWAS analysis was performed using the GEMMA software (http://www.xzlab.org/software.html (accessed on 20 January 2021)) which implements the Genome-wide Efficient Mixed Model Association algorithm [19]. Initially, a centered relatedness matrix was estimated for each color phenotype based on genotype. The association tests were then performed with the relatedness matrix, phenotype and genotype fit into a univariate linear mixed model. To reach genome-wide significance, Wald *p*-values from GEMMA had to pass a Bonferroni threshold at *p* < 0.05 calculated for 85,172 markers (genome-wide significance threshold: *p* = 5.9 × 10^−7^). Additionally, GEMMA reports PVE (“chip heritability”) which is an estimate of the proportion of phenotypic variance explained. The regression method in the R-package GenABEL was used to make estimates of λ [20]. Q-Q and Manhattan plots were generated through the qqman-package in R version 3.2.3. We checked whether there was any effect of age on overall “whiteness” score, by splitting the data into three age-classes and using a generalized linear model to analyze any possible differences but there was no such effect (Wald Chi-Square: 2.64; *p* = 0.27), so age was not included in the model.

Subsequent linkage disequilibrium (LD) analysis and visualization of haplotypes was done in the Haploview v4.2 software [21]. LD windows were estimated based upon solid spine of LD and SNPs that were considered to be linked had a D’ of 1 and LOD-score of >0.15. Visualization of the genomic position of significant SNPs in relation to CanFam3 mapped genes were done using the software Integrative Genomics Viewer (IGV) version 2.8.9 [22]. The association between genotypes of the most significant SNP for each phenotype was investigated using the non-parametric Kruskal–Wallis test in IBM SPSS Statistics 25. Pairwise comparisons were made, and Bonferroni was used to adjust p-values for multiple testing. A Spearman correlation was also carried out to investigate how the different phenotypic measures correlate with each other.

## 3. Results

The amount of white fur coverage of the face, neck, legs, body, and tail as well as black saddle coverage for each of the 190 beagles is listed in Supplementary Table S1. The inter-rater reliability analysis showed high agreement for all phenotypes (N = 19, Spearman Rho > 0.92, *p* < 0.001). Significant SNP-phenotype associations were found for the face, neck, legs, and overall white on a region on chromosome 20. The face and saddle coloration phenotypes were associated with a region on chromosome 5. A detailed description of our results is given below.

### 3.1. Color Phenotype

**Figure 1:**
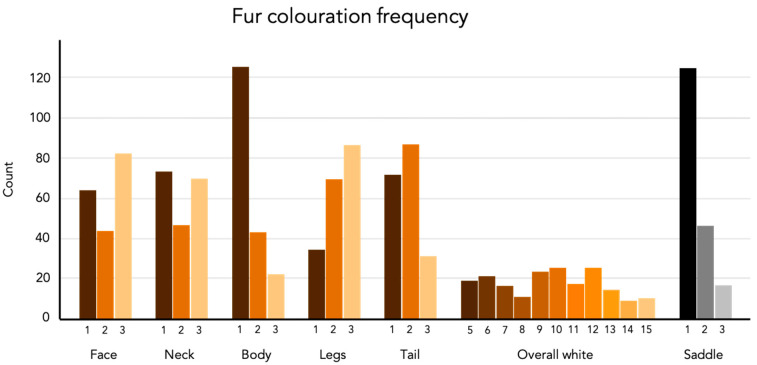
The frequency (count) of individuals for each phenotype scoring. The total number of individuals is 190 with Table 187 due to the exclusion of three dogs that did not express the saddle.

**Figure 2:**
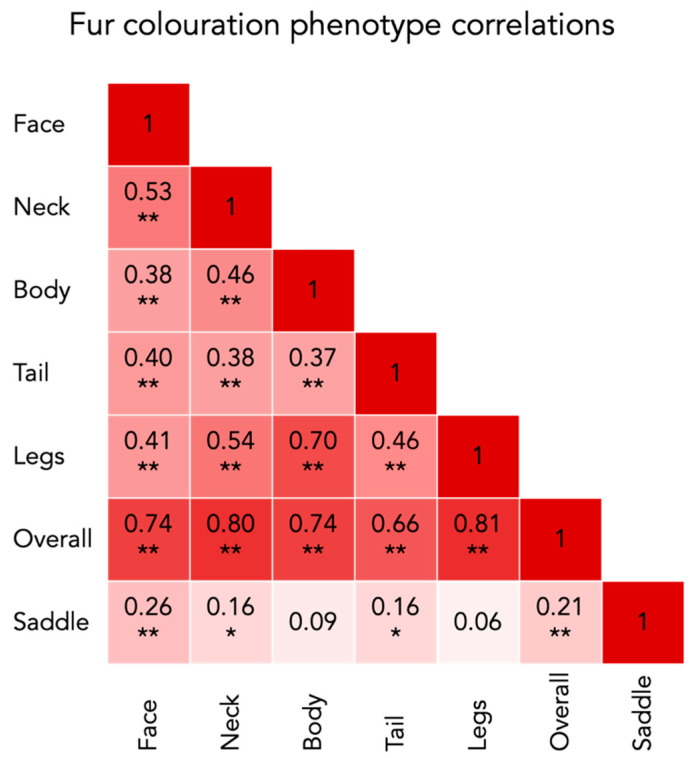
Spearman correlation of the amount of white in the fur coloration phenotypes. The boxes show the correlation coefficients with a corresponding color intensity. ** = *p* < 0.01; * = *p* < 0.05.

Frequencies of each phenotype are presented in Figure 1. A set of representative photographs of each of the phenotypes can be found in the supplementary material. There is a significant positive correlation between all of the white phenotypic measurements (Figure 2). This means that an individual with a high coverage of white patching on one body part typically have more white coverage on other areas as well. The saddle however, only correlated with the amount of white in the face and the combined measurement called overall white. The estimated proportion of variance explained (PVE) or “chip heritability” of the different fur coloration phenotypes ranged between 0.40 ± 0.13 (SE) for the coverage of white spotting in the face, to 0.79 ± 0.11 (SE) for the amount of white spotting covering the legs. All PVE estimates are presented in Table 2.

### 3.2. GWAS

After pruning the genotype data from uninformative and low-call rate markers, 85,172 SNPs remained for the GWAS analysis in GEMMA. The GEMMA software successfully accounted for population stratification as shown by the genomic inflation factor λ in Table 2 as well as the Q-Q plots in Supplementary Figure S1. The MDS plot is presented in Supplementary Figure S2.

**Figure 3:**
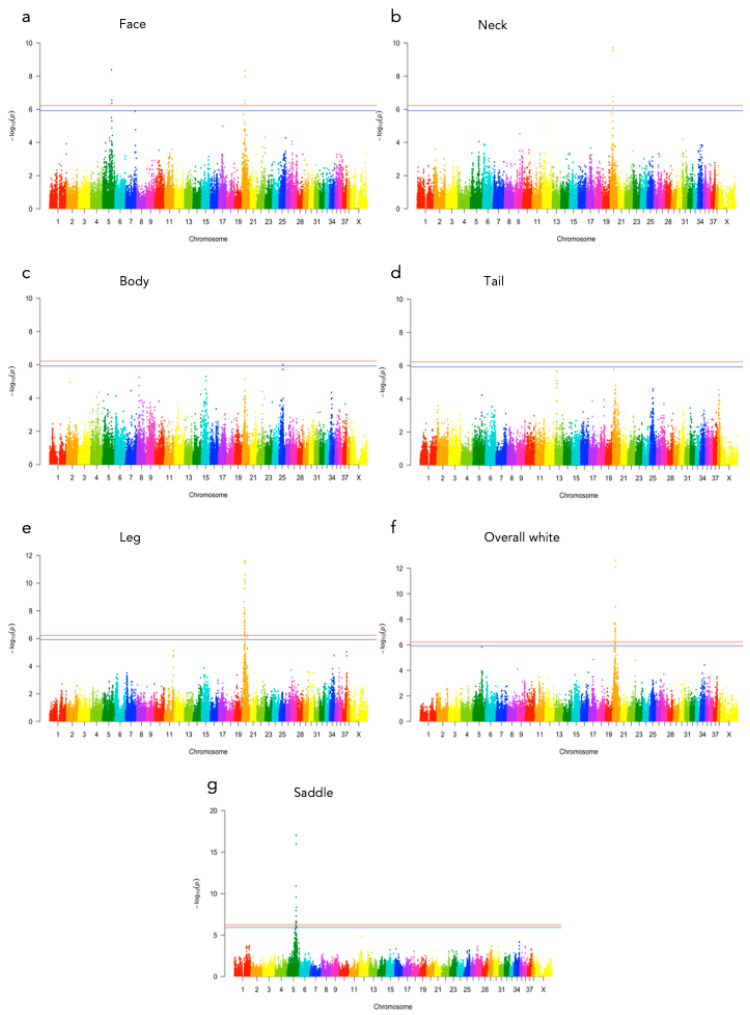
Manhattan plots of GEMMA p-values for each fur coloration phenotype (**a**) face, (**b**) neck, (**c**) body, (**d**) tail, (**e**) leg, (**f**) overall white and (**g**) saddle. Genome-wide significance levels are displayed as a red line (Bonferroni 5%) and a blue line (Bonferroni 10%).

**Figure 4:**
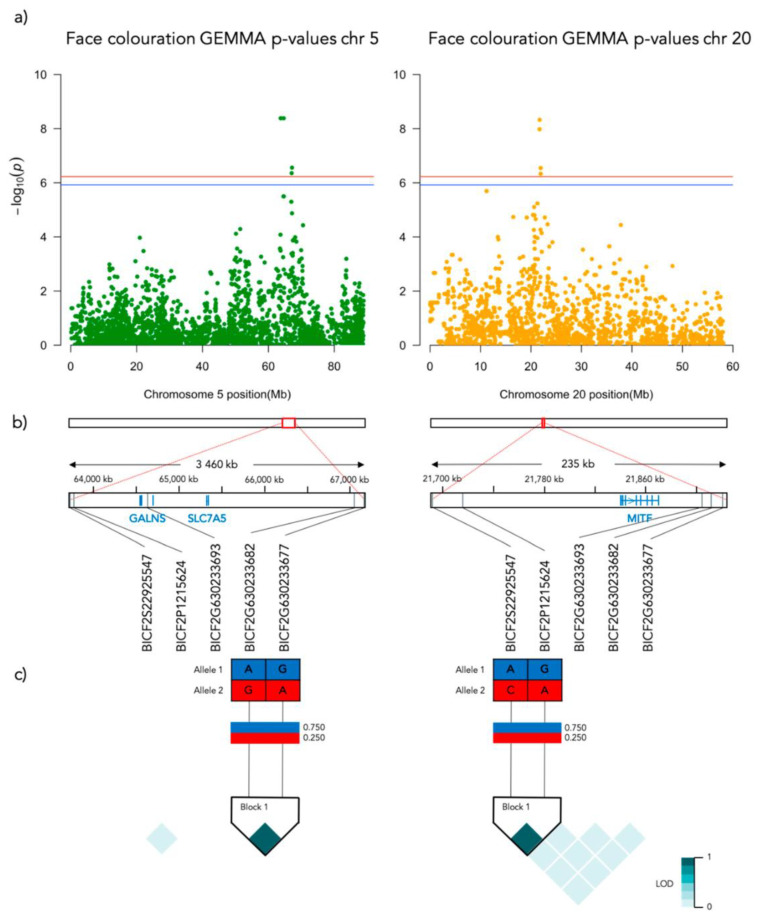
Results from the Genome-Wide Association analysis on the face coloration phenotype for chromosome 5 (left) and 20 (right). (**a**) Manhattan plot of the chromosomes with significantly associated markers. The red line represents genome-wide significance of Bonferroni 5% and the blue 10%. (**b**) (upper) A schematic figure of the size and positioning of the significant region on the chromosome. (lower) Zoomed figure of the associated region showing the positioning of the significant SNP markers in grey and genes in blue. (**c**) Linkage disequilibrium heat map and haplotype blocks of the significant markers.

**Figure 5:**
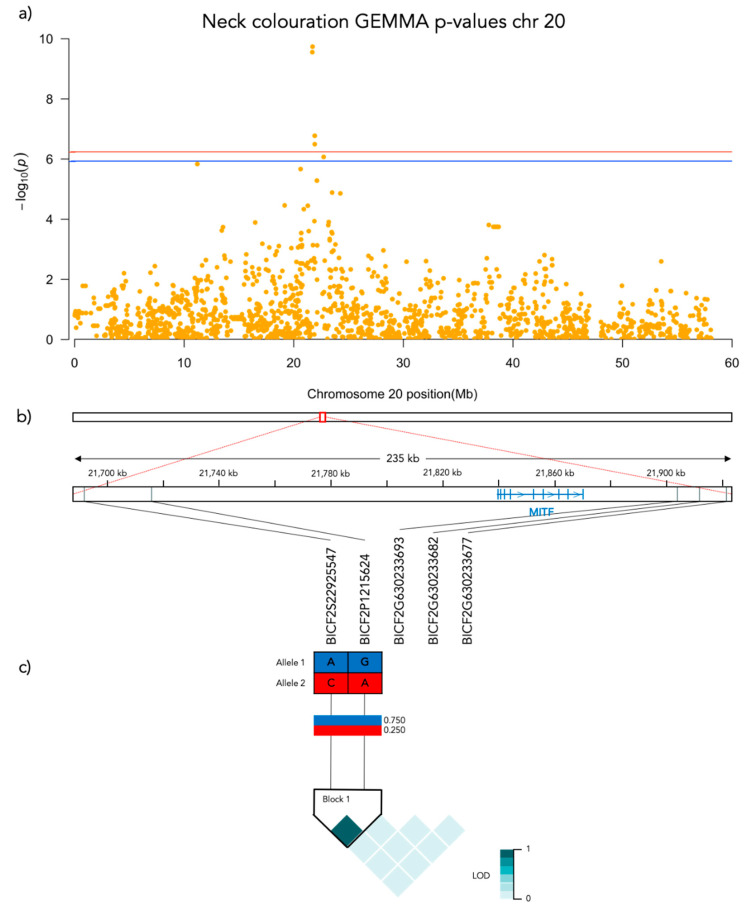
Results from the Genome-Wide Association analysis on the neck coloration phenotype for chromosome 20. (**a**) Manhattan plot of the chromosomes with significantly associated markers. The red line represents genome-wide significance of Bonferroni 5% and the blue 10%. (**b**) (upper) A schematic figure of the size and positioning of the significant region on the chromosome. (lower) Zoomed figure of the associated region showing the positioning of the significant SNP markers in grey and genes in blue. (**c**) Linkage disequilibrium heat map and haplotype blocks of the significant markers.

**Figure 6:**
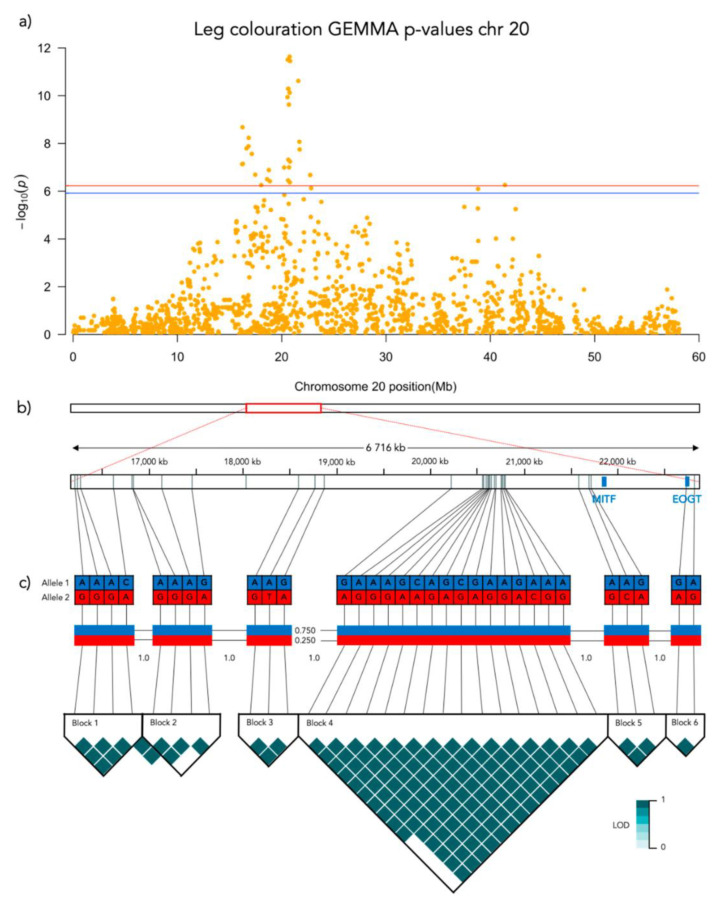
Results from the Genome-Wide Association analysis on the leg coloration phenotype for chromosome 20. (**a**) Manhattan plot of the chromosomes with significantly associated markers. The red line represents genome-wide significance of Bonferroni 5% and the blue 10%. (**b**) (upper) A schematic figure of the size and positioning of the significant region on the chromosome. (lower) Zoomed figure of the associated region showing the positioning of the significant SNP markers in grey and genes in blue. (**c**) Linkage disequilibrium heat map and haplotype blocks of the significant markers.

**Figure 7:**
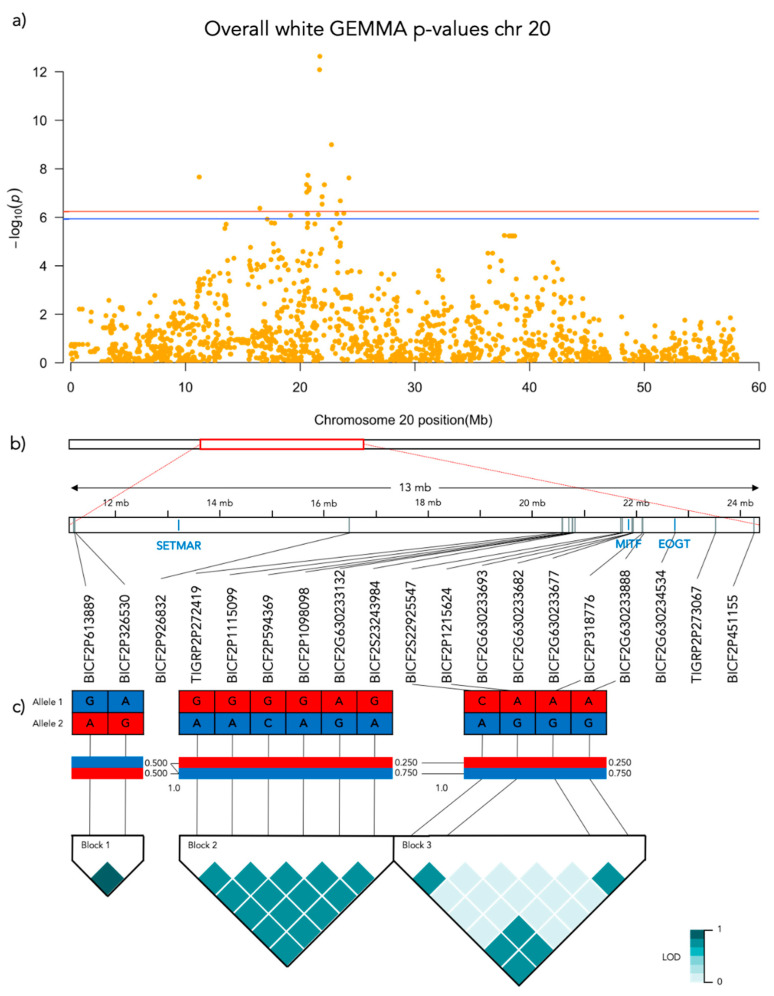
Results from the Genome-Wide Association analysis on the overall white phenotype for chromosome 20. (**a**) Manhattan plot of the chromosomes with significantly associated markers. The red line represents genome-wide significance of Bonferroni 5% and the blue 10%. (**b**) (upper) A schematic figure of the size and positioning of the significant region on the chromosome. (lower) Zoomed figure of the associated region showing the positioning of the significant SNP markers (grey) and genes (blue). (**c**) Linkage disequilibrium heat map and haplotype blocks of the significant markers.

**Figure 8:**
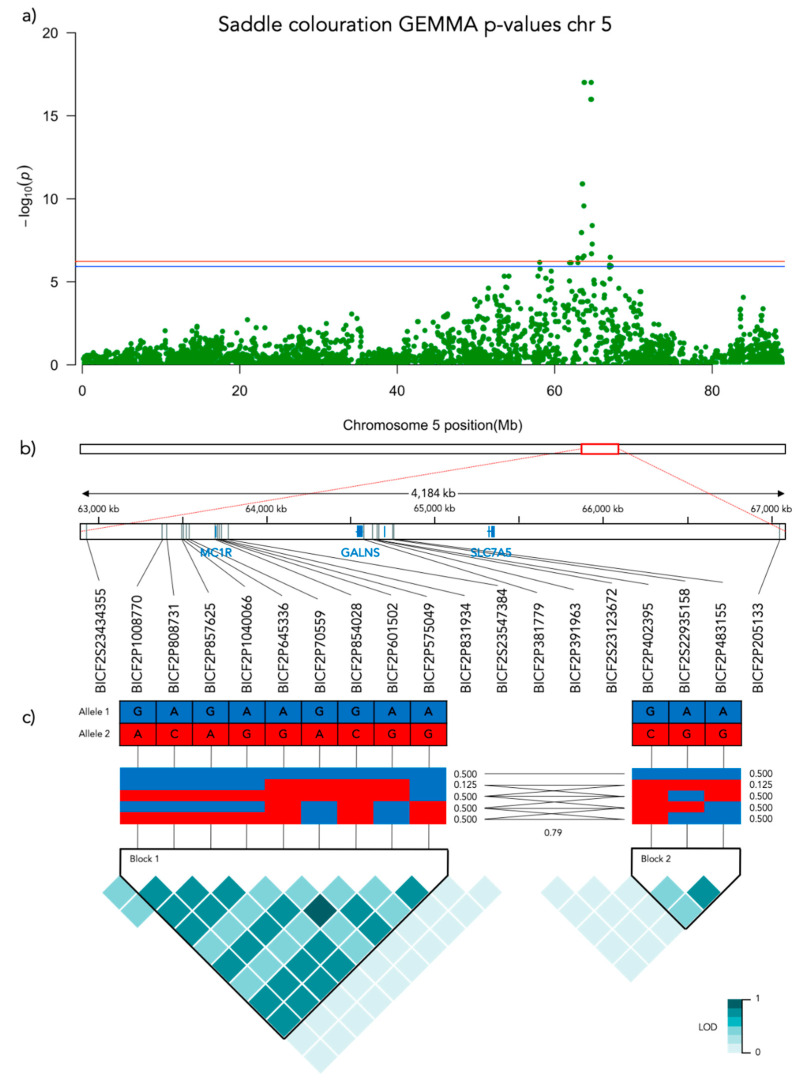
Results from the Genome-Wide Association analysis on the saddle coloration phenotype for chromosome 5. (**a**) Manhattan plot of the chromosomes with significantly associated markers. The red line represents genome-wide significance of Bonferroni 5% and the blue 10%. (**b**) (upper) A schematic figure of the size and positioning of the significant region on the chromosome. (lower) Zoomed figure of the associated region showing the positioning of the significant SNP markers in grey and genes in blue. (**c**) Linkage disequilibrium heat map and haplotype blocks of the significant markers.

Significant genotype associations were found for the amount of white coverage of the face, neck, legs, and overall white as well as the black pigmentation coverage of the saddle (Figure 3). No markers were found to be significantly associated with the amount of white fur covering the body and tail. The amount of white in the face is associated with a significant peak of 5 SNPs on chromosome 5 and 5 SNPs on chromosome 20 (Figure 4, Supplementary Table S1). The associated haplotypes do not overlap any gene regions, however, the face pigmentation associated markers are surrounding the region of the genes *GALNS* and *SLC7A5* on chromosome 5 and the *MITF*-gene on chromosome 20. The same 5 SNPs on chromosome 20, surrounding the *MITF*-gene, are also associated with white patching of the neck (Figure 5, Supplementary Table S2). Additionally, on chromosome 20 there is a peak of 34 SNP markers significantly associated with the amount of pigmented fur on the legs (Figure 6, Supplementary Table S3). The leg pigmentation-associated markers are linked in 6 haplotype blocks where block 6 is overlapping the *EOGT*-gene locus and the *MITF*-gene is positioned between the 5th and 6th block. Overall white is a combined score of all scored white patching phenotypes and this is significantly associated with 19 SNPs on chromosome 20 (Figure 7, Supplementary Table S4). The markers associated with overall white are linked in 3 associated haplotypes, where the 3rd one is overlapping the *MITF*-gene. Additionally, the marker BICF2G630234534 is positioned within the first of 14 introns of the *EOGT*-gene. The gene named *SETMAR* is positioned between the 1st and 2nd linkage blocks associated with overall white. The saddle phenotype is significantly associated with 19 SNP markers on chromosome 5 linked in two associated haplotypes (Figure 8, Supplementary Table S5). The 1st haplotype block is overlapping the region of the *MC1R*-gene. The gene *GALNS* is positioned between the two blocks while *EOGT* is positioned after the 2nd block.

### 3.3. Genotype Associations

#### 3.3.1. Face Coloration

**Figure 9:**
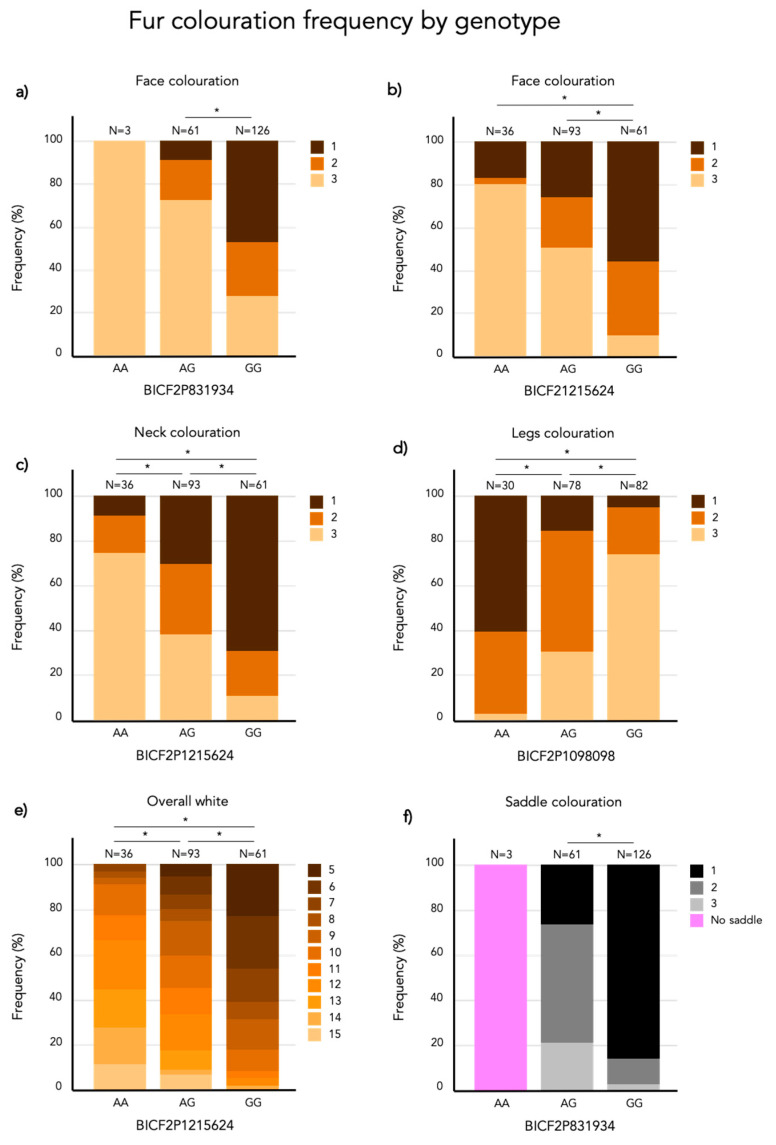
Stacked bar plot of the frequency (%) of each phenotype score per genotype of the most associated SNP marker for (**a**) face coloration (chr 5), (**b**) face coloration (chr 20), (**c**) neck coloration, (**d**) leg coloration, (**e**) overall white and (**f**) saddle coloration. Significant differences (*p* < 0.05) are marked with *. The numbers above each bar represents the total number of individuals carrying that genotype.

On chromosome 5, the three strongest markers associated with face coloration have the same Bonferroni adjusted *p*-value of 4.1 × 10^−9^. Figure 9a presents the frequency of face coloration for the different genotypes of the first of these three significant SNPs, BICF2P831934 positioned at chr5: 63728735 (N = 190, χ^2^ = 40.631, *p* < 0.001, df = 2). Face coloration frequencies differ significantly between GG and AG genotyped individuals (χ^2^ = 48.802, SE = 7.996, adj. *p* > 0.001). The face score frequencies for each genotype of the most associated SNP (BICF2P1215624, chr20: 21715930) on chromosome 20 (N = 190, χ^2^ = 42.625, *p* < 0.001, df = 2) is presented in Figure 9b. The face color frequency is significantly different between individuals with the GG and AG genotype (χ^2^ = 41.805, SE = 8.446, adj. *p* > 0.001) and GG and AA genotype (χ^2^ = 65.652, SE = 10.774, adj. *p* > 0.001).

#### 3.3.2. Neck Coloration

The marker with the strongest association (Bonferroni adjusted *p* = 1.9 × 10^−10^) with white patching of the neck is BICF2P1215624 at chr20: 21715930. There is a significant difference in neck coloration frequencies across the genotypes of BICF2P1215624 (N = 190, χ^2^ = 48.555, *p* < 0.001, df = 2; Figure 9c). In this case there is a significant difference between all pairwise compared genotypes: AA-GA (χ^2^ = 34.294, SE = 10.116, adj. *p* = 0.004), AA-GG (χ^2^ = 73.473, SE = 10.831, adj. *p* > 0.001) and AG-GG (χ^2^ = 39.179, SE = 8.491, adj. *p* > 0.001).

#### 3.3.3. Leg Coloration

White fur coverage of the legs was significantly associated with 34 SNPs on chromosome 20 where the strongest associated SNP is BICF2P1098098 (Bonferroni adjusted *p* = 2.3 × 10^−12^) positioned at chr20: 20754205. There frequency of leg coloration is significantly different across genotypes of BICF2P1098098 (N = 190, χ^2^ = 65.445, *p* < 0.001, df = 2; Figure 9d). All genotypes of BICF2P1098098 differed significantly in leg coloration in the pairwise comparison: AA-AG (χ^2^ = −44.600, SE = 10.902, adj. *p* > 0.001), AA-GG (χ^2^ = −84.088, SE = 10.828, adj. *p* > 0.001) and AG-GG (χ^2^ = −39.488, SE = 8.026, adj. *p* > 0.001).

#### 3.3.4. Overall White

Overall white is significantly associated with 19 SNP markers on chromosome 20 and the strongest association was found with the marker BICF2P1215624 (Bonferroni adjusted *p* = 2.4 × 10^−13^) positioned at chr20: 21715930. The frequency of overall white is significantly different across BICF2P1215624 genotypes (N = 190, χ^2^ = 65.429, *p* < 0.001, df = 2; Figure 9e). All genotypes of BICF2P1215624 differed significantly in leg coloration in the pairwise comparison: GG-GA (χ^2^ = 50.998, SE = 9.011, adj. *p* > 0.001), GG-AA (χ^2^ = 89.316, SE = 11.495, adj. *p* > 0.001) and GA-AA (χ^2^ = 38.318, SE = 10.736, adj. *p* = 0.002).

#### 3.3.5. Saddle Coloration

The amount of black coloration of the saddle is significantly associated with 19 SNPs on chromosome 5, where the strongest association was found with three SNPs that share the same Bonferroni-adjusted *p*-value of 9.8 × 10^−18^. These are the same three SNP markers on chromosome 5 that also had the strongest association with face coloration. Figure 9f presents the frequency of saddle coloration for the different genotypes of the first of these three significant SNPs, BICF2P831934 positioned at chr5: 63728735 (N = 187, χ^2^ = 64.129, *p* < 0.001, df = 2). No pairwise comparisons were made as there are only two genotypes, AG and GG in the model. Note that 187 individuals were included in the GWAS of the saddle phenotype as 3 individuals did not seem to display any black pigments. However, these three individuals turned out to be the only carriers of the AA-genotype at BICF2P831934. To acknowledge this, they were included in Figure 9f but not in the statistical analysis.

## 4. Discussion

This genome-wide association study used a high-density canine SNP chip to identify two genomic regions associated with the variation in loss of pigmentation in a population of piebald saddle-tan beagles. The dogs were scored based on the amount of white patching covering the face, neck, body, tail, legs, overall white, and the amount of solid black fur coverage of the saddle. White patching of the face, neck, legs, and overall white was associated with a region on chromosome 20, overlapping the *MITF*-gene locus positioned at 21612927-21870578 (CanFam3.1). Additionally, the variation in face and saddle coloration was associated with a region on chromosome 5 overlapping the locus of the *MC1R* gene. Both these genes have previously been associated with fur coloration phenotypes, although the mechanisms are still insufficiently known. The associations demonstrated in the present paper suggest that regulatory mutations may affect loss of pigmentation of different parts of the body in beagles, and therefore adds significant knowledge to the quantitative variation in fur coloration in dogs. Future research should be able to determine the causative mutations by genotyping known variants or DNA-sequencing of individuals with contrasting phenotypes.

The temporal and spatial production of pigments is controlled by the interaction of multiple genes. The current state of knowledge with respect to the genetics of fur coloration in dogs has recently been extensively reviewed [8]. In this paper, we focused on one breed, the beagle, that is typically 3-colored as it has the saddle-tan phenotype with piebald white spotting. Piebald white patching has previously been mapped to the *MITF*-gene [23,24]. To express piebald spotting, the dog needs to carry two copies of the *s^p^*-allele of the gene *microphthalmia-associated transcription factor* (*MITF*) [13,25]. Beagles are fixed for the piebald white spotting *s^p^*-allele at the *MITF*-gene [12] but yet we see a significant amount of variation in the amount of white fur coverage. The chip heritability of this variation ranged between 0.40 and 0.79 indicating that there is a significant heritable genetic basis for this variation in piebald white patching in the studied beagle population. Additionally, all of the variation in white patching phenotypes in face, neck, body, tail, and legs were significantly positively correlated while there was a considerably smaller correlation between saddle and the rest of the body. This raises the possibility that the same genetic mechanisms underlie most of the white patching except for the saddle, where other or additional mechanisms may play a role.

Since *MITF* is regulated in a highly complex manner, including several different start codons and with different functions in different tissues, it is most likely that the phenotypic variations are a result of regulatory differences between individuals. Consistent with this, it has been found that variations in the regulation of *MITF* by means of a simple repeat polymorphism in the *MITF-M* promoter may explain the degree of white spotting in a variety of dog breeds [13]. In the present study, we found that loss of pigmentation on leg, body, neck, and face was associated with SNP markers closely associated with the *MITF*-gene, but also with adjacent genes in the same haplotype blocks such as *EOGT* and *SETMAR*. *EOGT* is an N-acetylglucosamine transferase and *SETMAR* encodes a histone-lysine N-methyltransferase that may be involved in the methylation of H3 histones. Because of the linkage blocks in this population, we cannot assess which of the associations that are putatively causative, but the results suggest that the variations in phenotype may possibly be related to regulatory variants associated with several genes in this haplotype block.

With respect to the saddle, we found a strong association with a region on chromosome 5, covering *MC1R*, with *GALNS* and *SLC7A5* in the same haplotype block. Three genes have previously been suggested to be involved in the expression of the saddle tan phenotype, i.e., *ASIP*, *RALY,* and *MC1R* but recent research has convincingly demonstrated that *RALY* is most likely not involved in regulating pigmentation [10]. Saddle tan requires that the dog has the *a^t^* (tan points) allele of the *ASIP* (*Agouti Signaling Protein*) that is also associated with the tan points or black-and-tan phenotype [14]. In this study, we did not find any associations with *ASIP*, which could possibly indicate that the beagles studied here were fixed on this allele.

The expression of the saddle tan phenotype is also relying on the ability of the dog to produce black pigment. Hence, if the dog is homozygous for the *e*-allele (recessive red) of the *MC1R*-gene it will only be able to express a pheomelaninistic phenotype (no black pigments) [26,27]. This could possibly cause the lack of black pigment in our three beagles excluded from the analysis of the saddle coloration due to the lack of a saddle. It is possible that the A-allele at the BICF2P831934 SNP locus could be linked to the recessive red variant of the *MC1R* gene. Although speculative, this is supported by the fact that beagles heterozygous at this locus have a significantly more faded saddle than those carrying the GG-genotype and express a more solid black saddle.

The melanocortin 1 receptor (*MC1R*) controls which type of melanin that can be produced by melanocytes, and when activated stimulates the cells to produce eumelanin. At least three alleles have been described in dogs, where loss-of-function mutations cause an increased production of phaeomelanin, leading to red coat color [7]. It is not clear in what way the association we have found can be explained, i.e., by which mechanisms *MC1R* could affect the degree of whiteness. However, similar associations (based on hereditary patterns) have been reported in horses, where genotype on the E-locus (*MC1R*) has been linked to the quantitative expression of white markings in the face region [28]. It is therefore possible that whiteness is associated with regulatory mutations affecting expression levels of *MC1R* and also that complex’s interactions with other genes may play a role. For example, the *KIT* gene (dominant white locus) is related to white spotting in many species, [29]. It encodes the tyrosine kinase receptor and interacts reciprocally with *MITF* but has not been implicated to play a role in relation to *MC1R*, and in fact, its effect on whiteness in dogs is unclear [29].

It is of course important to remember that we have studied only one specific population of beagles, and the alleles present in this population may not be representative for all other beagles. Hence, several other alleles may exist in the breed, and some of them may have subtle functional differences and may therefore also have been overlooked in the classical studies of Little from 1957 [9].

In conclusion, we find that the complex variation in loss of pigmentation in beagles, including the quantitative variation in expression of the saddle, can be attributed to the genetic variation on mainly two loci. These loci contain two genes, previously found to be associated with pigmentation, namely *MC1R* and *MITF,* although other genes are also linked to significant markers associated with the phenotypes. Our results suggest that the large variation in pigmentation patterning may possibly be a function of the regulatory control of *MC1R* and *MITF* genes.

## Figures and Tables

**Table 1:** Fur coloration scoring. A description of the scoring criteria for the different fur pigmentation phenotypes. Overall white is a combined score of all white patching phenotypes.

Phenotype	Score	Name	Description
Face	1	No or residual white	Solid colored face with no, or only, residual white around the nose.
	2	White muzzle	White markings around the nose, partly or completely extending over the muzzle, no blaze but a thin streak may occur.
	3	Blaze	As described above but also including a blaze reaching to or above eye level.
Neck	1	No white	Except for the ventral side, solid colored neck.
	2	Residual white	Except for the ventral side, small residual white patches or thin stripes around the neck.
	3	Collar	The white on the ventral side of the neck extends as a collar towards or around the dorsal side. Large patches.
Body	1	Solid	No or only residual white patches on the body except for the neck area and the ventral side.
	2	Some white	Only one or two smaller white patch/patches on the body except for the neck area and the ventral side, usually extending from the hind legs.
	3	White patches	Large white patches, usually on both sides of the body.
Legs	1	Half white	Legs are less white than and does not fulfil the criteria of score 2.
	2	Mostly white	At least 3 legs are between 2 and 3 to fully white.
	3	Completely white	At least 3 legs fully white and the white extends from the leg on to the body from at least one leg.
Tail	1	White tip	1/4 or less of the tail is white.
	2	Half white	More than 1/4 but less than 3/4 of the tail is white.
	3	Mostly white	The tail is more than 3/4 white.
Overall white	5–15		The sum of scores from face, neck, body, legs and tail.
Saddle	1	Solid	Solid black saddle from the neck to the tail base.
	2	Semi-faded	Mostly solid black saddle from the neck to the tail base, but brown patches occurs.
	3	Faded	No or mostly no solid black saddle.

**Table 2:** Proportion of variance explaine (PVE) or “chip heritability” and λ (genomic inflation factor) from the GWAS analysis in GEMMA.

	Face	Neck	Body	Legs	Tail	Overall	Saddle
PVE	0.40	0.59	0.55	0.79	0.46	0.77	0.61
SE (PVE)	0.13	0.15	0.14	0.11	0.14	0.11	0.19
λ	1.07	1.05	1.11	1.08	1.04	1.09	1.09
SE (λ)	0.0004	0.0003	0.0002	0.0009	0.0002	0.0007	0.001

## Data Availability

Genotype data is publicly available at https://osf.io/m5a3v/?view_only=a6e3a4383f894d7cbda34c0750f26fb0 (accessed on 10 January 2021).

## Supplementary Materials

The following are available online at https://www.mdpi.com/2073-4425/12/2/316/s1, Figure S1: Q_Q plots, Figure S2: MDS plot of population structure, Table S1: SNP markers face, Table S2: SNP markers neck, Table S3: SNP markers leg, Table S4: SNP markers overall white, Table S5: SNP markers saddle, Photos S1: Photos of representative phenotypes.
