# Supplementary material for: Genomic Regions Associated with Variation in Pigmentation Loss in Saddle Tan Beagles
Source: Genes (Basel). 2021 Feb 23;12(2):316. doi: 10.3390/genes12020316 (PMC7926638; doi:10.3390/genes12020316)
Supplement: Supplementary file 1 [file genes-12-00316-s001.zip › Zip suppl/ColourGWASSupplementary201029.docx]

Supplementary figures and tables: Nord and Jensen, Genomic regions associated with variation in fur coloration in saddle tan beagles


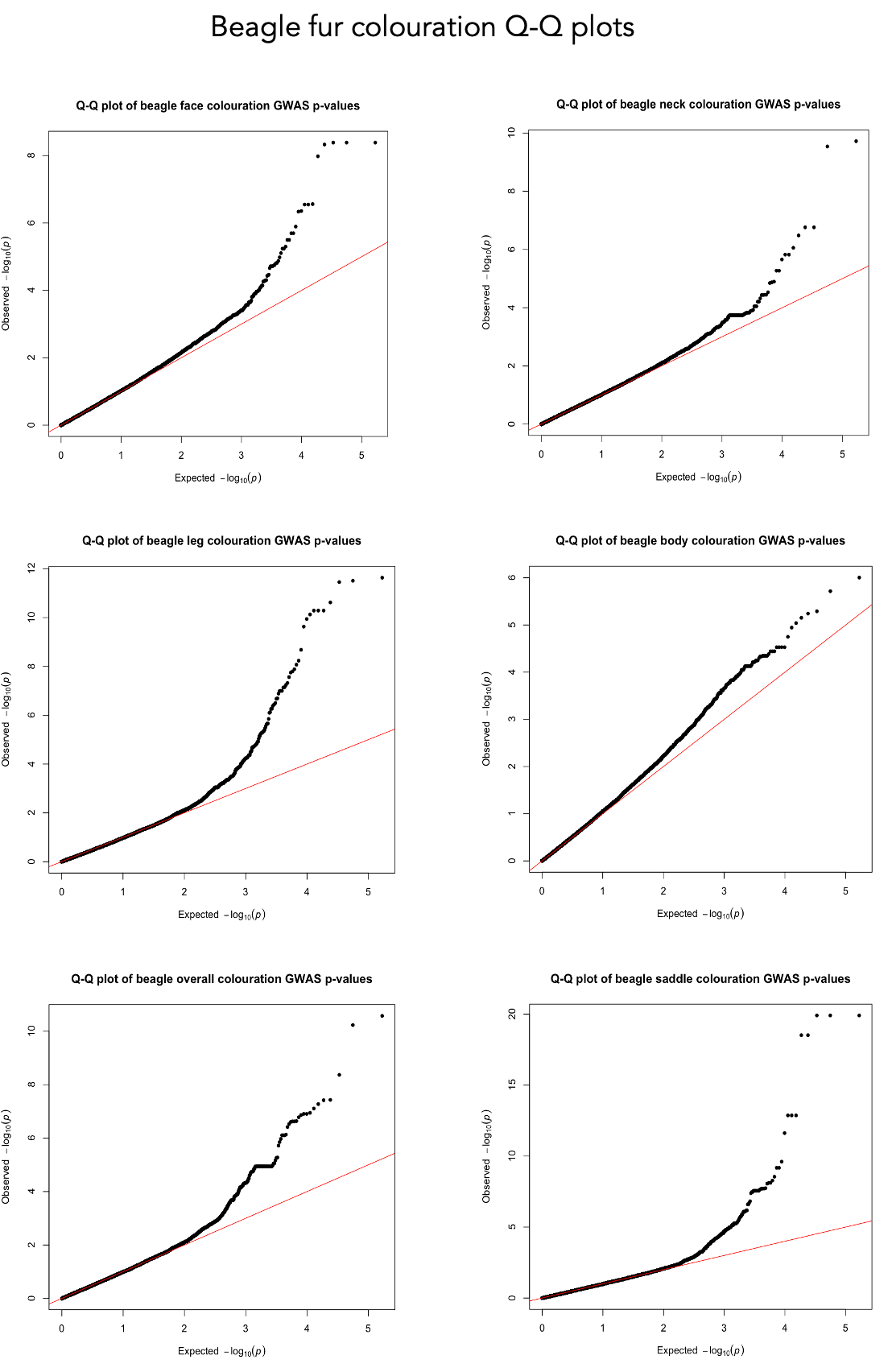


Supplementary Figure 1: Q-Q plots of GEMMA Wald p-values.


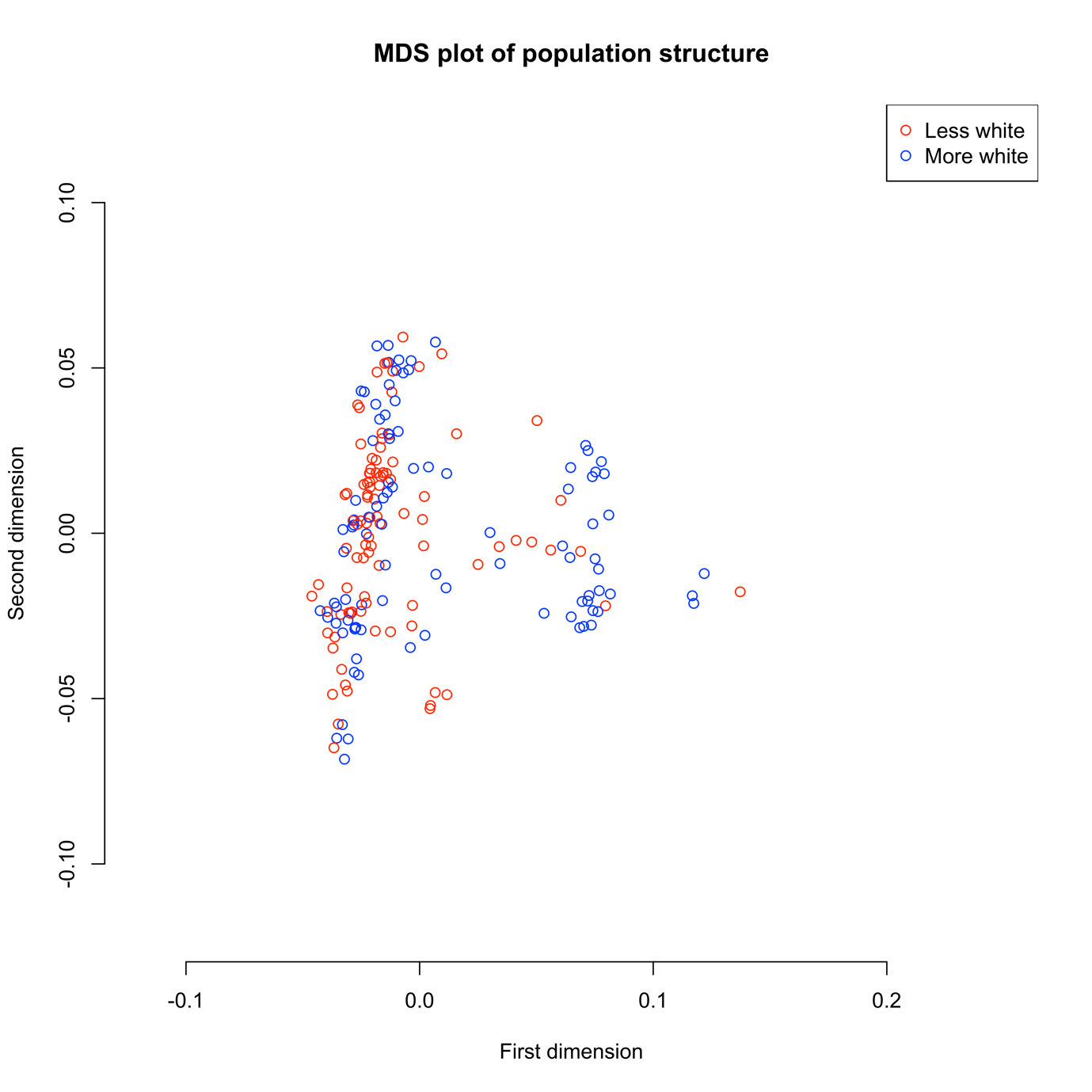


Supplementary Figure 2: Multidimentional scaling plot of the first and second dimention for the studied population of 190 beagles. Red dots represent the 95 most white dogs while the blue dots represents the 95 least white dogs.

Supplementary Table 1: SNP markers significantly associated with variation in face colouration. The linkage blocks are visualised in Figure 4.

| SNP | CHR | BP | P (Wald) | Haplo-block | Haploblock size (kb) | Haploblock range | Genes |
| --- | --- | --- | --- | --- | --- | --- | --- |
| BICF2S22925547 | 20 | 21691979 | 1.049e-08 | 1 | 24 | 21691979-21715930 |  |
| BICF2P1215624 | 20 | 21715930 | 4.708e-09 | 1 | 23 | 21691979-21715930 |  |
| BICF2G630233693 | 20 | 21904057 | 2.840e-07 |  |  |  |  |
| BICF2G630233682 | 20 | 21911990 | 4.625e-07 |  |  |  |  |
| BICF2G630233677 | 20 | 21920199 | 2.840e-07 |  |  |  |  |
| BICF2P831934 | 5 | 63728735 | 4.129e-09 |  |  |  |  |
| BICF2S23547384 | 5 | 63770156 | 4.129e-09 |  |  |  |  |
| BICF2P391963 | 5 | 64630776 | 4.129e-09 |  |  |  |  |
| BICF2P205133 | 5 | 67050262 | 4.412e-07 | 1 | 111 | 67050262-67161387 |  |
| BICF2G630242040 | 5 | 67161387 | 2.763e-07 | 1 | 111 | 67050262-67161387 |  |

Supplementary Table 2: SNP markers significantly associated with variation in neck colouration. The linkage blocks are visualised in Figure 5.

| SNP | CHR | BP | P (Wald) | Haplo-block | Haploblock size (kb) | Haploblock range | Genes |
| --- | --- | --- | --- | --- | --- | --- | --- |
| BICF2S22925547 | 20 | 21691979 | 2.902e-10 | 1 | 23 | 21691979-21715930 |  |
| BICF2P1215624 | 20 | 21715930 | 1.903e-10 | 1 | 23 | 21691979-21715930 |  |
| BICF2G630233693 | 20 | 21904057 | 1.729e-07 |  |  |  |  |
| BICF2G630233682 | 20 | 21911990 | 3.302e-07 |  |  |  |  |
| BICF2G630233677 | 20 | 21920199 | 1.729e-07 |  |  |  |  |

Supplementary Table 3: SNP markers significantly associated with variation in leg colouration. The linkage blocks are visualised in Figure 6.

| SNP | CHR | BP | P (Wald) | Haplo-  block | Haploblock size (kb) | Haploblock range | Genes | | |  |
| --- | --- | --- | --- | --- | --- | --- | --- | --- | --- | --- |
| BICF2P483847 | 20 | 16205677 | 7.347e-08 | 1 | 417 | 16205677-16623190 | |  | |  |
| BICF2P492349 | 20 | 16240738 | 2.093e-09 | 1 | 417 | 16205677-16623190 | |  | |  |
| BICF2P1372858 | 20 | 16272832 | 7.083e-08 | 1 | 417 | 16205677-16623190 | |  | |  |
| BICF2S23615297 | 20 | 16623190 | 1.606e-08 | 1 | 417 | 16205677-16623190 | |  | |  |
| BICF2P253312 | 20 | 16817313 | 1.304e-08 | 2 | 314 | 16817313-17132311 | |  | |  |
| BICF2P1144936 | 20 | 16834026 | 5.808e-09 | 2 | 314 | 16817313-17132311 | |  | |  |
| BICF2P255275 | 20 | 17132311 | 2.730e-08 | 2 | 314 | 16817313-17132311 | |  | |  |
| BICF2G630230266 | 20 | 17458417 | 2.030e-07 |  |  |  |  | |  | |
| BICF2G630230774 | 20 | 18037927 | 5.516e-07 |  |  |  | | | |  |
| BICF2P850355 | 20 | 18594112 | 3.150e-07 | 3 | 270 | 18594112-18864380 | |  | |  |
| BICF2S23721855 | 20 | 18768767 | 1.300e-07 | 3 | 270 | 18594112-18864380 | |  | |  |
| BICF2G630231444 | 20 | 18864380 | 3.811e-07 | 3 | 270 | 18594112-18864380 | |  | |  |
| BICF2G630232660 | 20 | 20226838 | 1.016e-07 | 4 | 575 | 20226838-20802227 | |  | |  |
| TIGRP2P272419  _rs8893890 | 20 | 20555326 | 1.143e-10 | 4 | 575 | 20226838-20802227 | |  | |  |
| BICF2P1115099 | 20 | 20569369 | 3.095e-12 | 4 | 575 | 20226838-20802227 | |  | |  |
| BICF2G630233039 | 20 | 20605348 | 5.145e-11 | 4 | 575 | 20226838-20802227 | |  | |  |
| BICF2G630233041 | 20 | 20606862 | 3.514e-07 | 4 | 575 | 20226838-20802227 | |  | |  |
| BICF2G630233057 | 20 | 20622687 | 5.145e-11 | 4 | 575 | 20226838-20802227 | |  | |  |
| BICF2G630233071 | 20 | 20629558 | 4.759e-08 | 4 | 575 | 20226838-20802227 | |  | |  |
| BICF2G630233096 | 20 | 20651866 | 5.145e-11 | 4 | 575 | 20226838-20802227 | |  | |  |
| BICF2P594369 | 20 | 20682992 | 2.344e-10 | 4 | 575 | 20226838-20802227 | |  | |  |
| BICF2G630233108 | 20 | 20694427 | 9.975e-08 | 4 | 575 | 20226838-20802227 | |  | |  |
| BICF2G630233110 | 20 | 20699604 | 9.975e-08 | 4 | 575 | 20226838-20802227 | |  | |  |
| BICF2P1098098 | 20 | 20754205 | 2.315e-12 | 4 | 575 | 20226838-20802227 | |  | |  |
| BICF2G630233132 | 20 | 20756528 | 7.444e-11 | 4 | 575 | 20226838-20802227 | |  | |  |
| BICF2P1178111 | 20 | 20771917 | 4.239e-07 | 4 | 575 | 20226838-20802227 | |  | |  |
| BICF2G630233142 | 20 | 20790427 | 5.728e-08 | 4 | 575 | 20226838-20802227 | |  | |  |
| BICF2S23243984 | 20 | 20802227 | 3.524e-12 | 4 | 575 | 20226838-20802227 | |  | |  |
| BICF2P616686 | 20 | 21585331 | 2.398e-11 | 5 | 130 | 21585331-21715930 | |  | |  |
| BICF2S22925547 | 20 | 21691979 | 8.494e-09 | 5 | 130 | 21585331-21715930 | |  | |  |
| BICF2P1215624 | 20 | 21715930 | 1.787e-08 | 5 | 130 | 21585331-21715930 | |  | |  |
| BICF2G630234534 | 20 | 22726919 | 2.108e-07 | 6 | 94 | 22726919-22821388 | EOGT | |  | |
| BICF2P1100420 | 20 | 22821388 | 2.108e-07 | 6 | 94 | 22726919-22821388 | EOGT | |  | |
| BICF2P1394766 | 20 | 41401124 | 5.478e-07 |  |  |  |  | |  | |

Supplementary Table 4: SNP markers significantly associated with variation in overall white. The linkage blocks are visualised in Figure 7.

| SNP | CHR | BP | P (Wald) | Haplo-block | Haploblock size (kb) | Haploblock range | Genes |
| --- | --- | --- | --- | --- | --- | --- | --- |
| BICF2P613889 | 20 | 11195510 | 2.264e-08 | 1 | 12 | 11195510-11208465 |  |
| BICF2P326530 | 20 | 11208465 | 2.264e-08 | 1 | 12 | 11195510-11208465 |  |
| BICF2P926832 | 20 | 16480874 | 4.383e-07 |  |  |  |  |
| TIGRP2P272419  _rs8893890 | 20 | 20555326 | 4.631e-08 | 2 | 246 | 20555326-20802227 | MITF |
| BICF2P1115099 | 20 | 20569369 | 9.607e-08 | 2 | 246 | 20555326-20802227 | MITF |
| BICF2P594369 | 20 | 20682992 | 1.918e-08 | 2 | 246 | 20555326-20802227 | MITF |
| BICF2P1098098 | 20 | 20754205 | 7.982e-08 | 2 | 246 | 20555326-20802227 | MITF |
| BICF2G630233132 | 20 | 20756528 | 7.480e-08 | 2 | 246 | 20555326-20802227 | MITF |
| BICF2S23243984 | 20 | 20802227 | 6.166e-08 | 2 | 246 | 20555326-20802227 | MITF |
| BICF2S22925547 | 20 | 21691979 | 8.582e-13 | 3 | 424 | 21691979-22116909 | MITF |
| BICF2P1215624 | 20 | 21715930 | 2.418e-13 | 3 | 424 | 21691979-22116909 | MITF |
| BICF2G630233693 | 20 | 21904057 | 1.464e-07 |  |  |  |  |
| BICF2G630233682 | 20 | 21911990 | 2.979e-07 |  |  |  |  |
| BICF2G630233677 | 20 | 21920199 | 1.464e-07 |  |  |  |  |
| BICF2P318776 | 20 | 22104719 | 4.718e-08 | 3 | 424 | 21691979-22116909 |  |
| BICF2G630233888 | 20 | 22116909 | 4.718e-08 | 3 | 424 | 21691979-22116909 |  |
| BICF2G630234534 | 20 | 22726919 | 1.051e-09 |  |  |  | EOGT (intron 1/14) |
| TIGRP2P273067  _rs8691722 | 20 | 23501978 | 2.172e-07 |  |  |  |  |
| BICF2P451155 | 20 | 24250039 | 2.465e-08 |  |  |  |  |

Supplementary Table 5: SNP markers significantly associated with variation in saddle colouration. The linkage blocks are visualised in Figure 8.

| SNP | CHR | BP | P (Wald) | Haplo-block | Haploblock size (kb) | Haploblock range | Genes |
| --- | --- | --- | --- | --- | --- | --- | --- |
| BICF2S23434355 | 5 | 62931527 | 3.640e-07 |  |  |  |  |
| BICF2P1008770 | 5 | 63383195 | 1.083e-08 | 1 | 334 | 63383195-63718071 | MC1R |
| BICF2P808731 | 5 | 63406809 | 1.083e-08 | 1 | 334 | 63383195-63718071 | MC1R |
| BICF2P857625 | 5 | 63498067 | 1.263e-11 | 1 | 334 | 63383195-63718071 | MC1R |
| BICF2P1040066 | 5 | 63506496 | 1.263e-11 | 1 | 334 | 63383195-63718071 | MC1R |
| BICF2P645336 | 5 | 63520829 | 3.640e-07 | 1 | 334 | 63383195-63718071 | MC1R |
| BICF2P70559 | 5 | 63539215 | 1.263e-11 | 1 | 334 | 63383195-63718071 | MC1R |
| BICF2P854028 | 5 | 63697949 | 2.695e-10 | 1 | 334 | 63383195-63718071 | MC1R |
| BICF2P601502 | 5 | 63710280 | 2.791e-07 | 1 | 334 | 63383195-63718071 | MC1R |
| BICF2P575049 | 5 | 63718071 | 2.791e-07 | 1 | 334 | 63383195-63718071 | MC1R |
| BICF2P831934 | 5 | 63728735 | 9.782e-18 |  |  |  |  |
| BICF2S23547384 | 5 | 63770156 | 9.782e-18 |  |  |  |  |
| BICF2P381779 | 5 | 64579861 | 1.026e-16 |  |  |  |  |
| BICF2P391963 | 5 | 64630776 | 9.782e-18 |  |  |  |  |
| BICF2S23123672 | 5 | 64662160 | 1.026e-16 |  |  |  |  |
| BICF2P402395 | 5 | 64666164 | 2.064e-07 | 2 | 89 | 64666164-64755545 |  |
| BICF2S22935158 | 5 | 64754407 | 4.107e-09 | 2 | 89 | 64666164-64755545 |  |
| BICF2P483155 | 5 | 64755545 | 5.355e-08 | 2 | 89 | 64666164-64755545 |  |
| BICF2P205133 | 5 | 67050262 | 3.349e-07 |  |  |  |  |
