# Supplementary material for: Genomic Regions Associated with Variation in Pigmentation Loss in Saddle Tan Beagles
Source: Genes (Basel). 2021 Feb 23;12(2):316. doi: 10.3390/genes12020316 (PMC7926638; doi:10.3390/genes12020316)

Nord & Jensen: Genomic regions associated with variation in fur coloration in saddle tan beagles

Supplementary material: Representative pictures of dogs with different colour phenotypes.

Face

Score	Name	Description
1	No or residual white	Solid coloured face with no, or only, residual white around the nose
2	White muzzle	White markings around the nose, partly or completely extending over the muzzle, blaze not reaching to or above eye level
3	Blaze	As described above but also including a blaze reaching to or above eye level

1

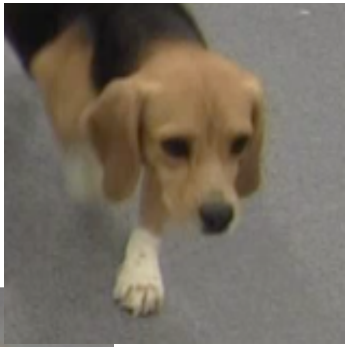
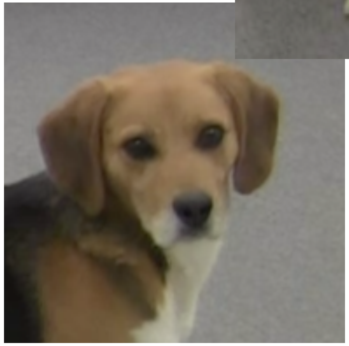

2

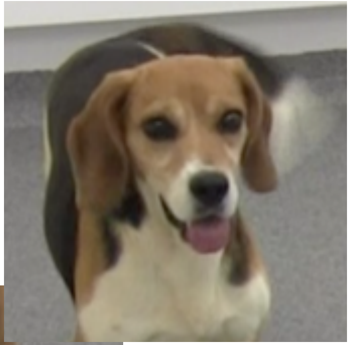
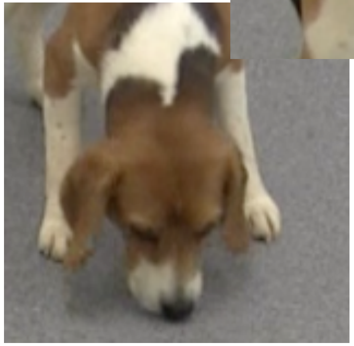

3

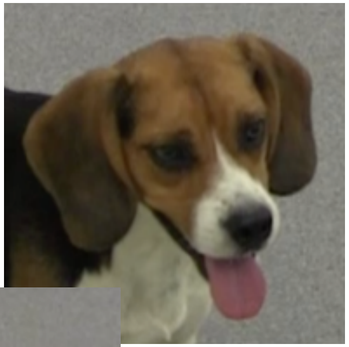
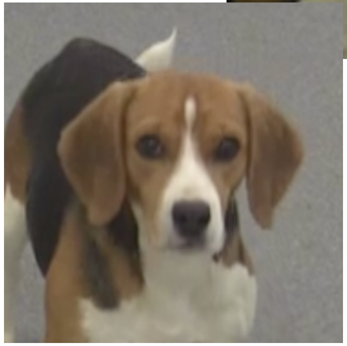

Neck

Score	Name	Description
1	No white	Except for the ventral side, solid coloured neck
2	Residual white	Except for the ventral side, small residual white patches or thin stripes around the neck.
3	Collar	The white on the ventral side of the neck extends as a collar towards or around the dorsal side. Large patches.

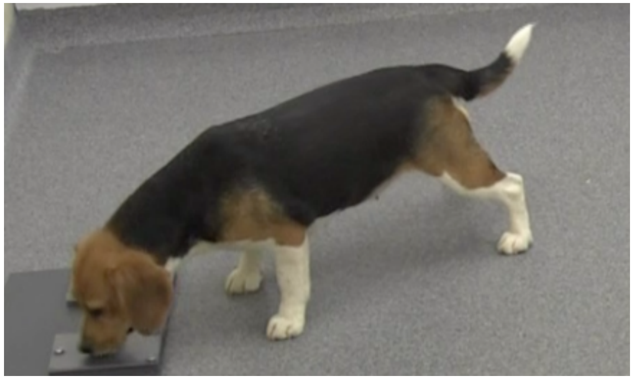

1

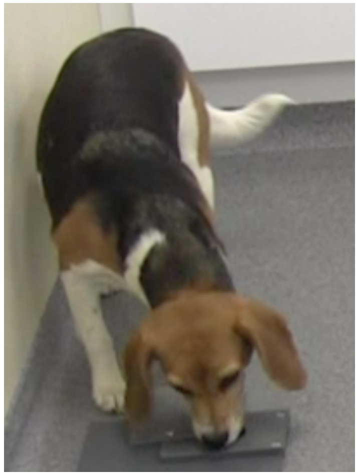

2

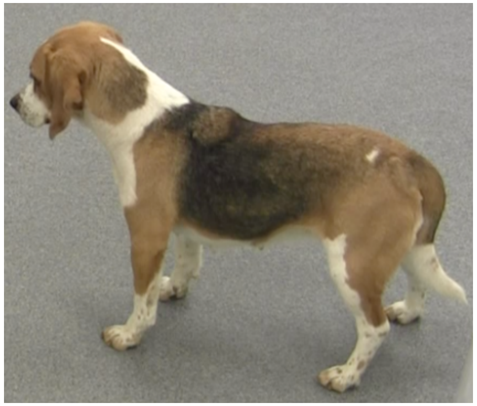

3

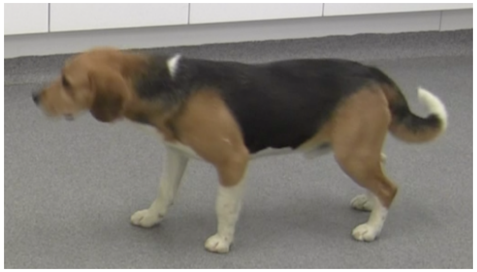

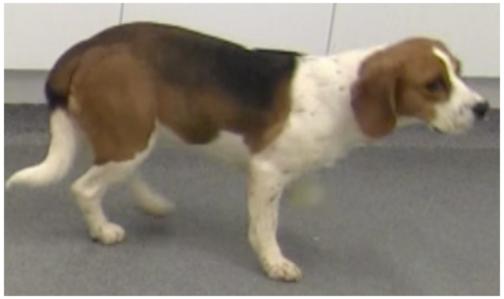

Tail

Score	Name	Description
1	Little white	25% or less white tail
2	Half white	About half of the tail is white, >25%, <75%
3	Mostly white	75% or more white tail

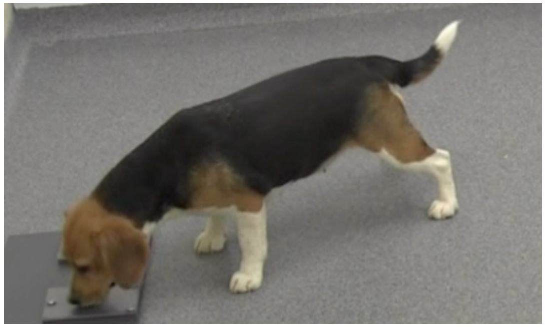

1

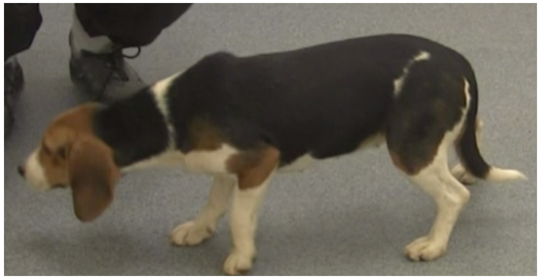

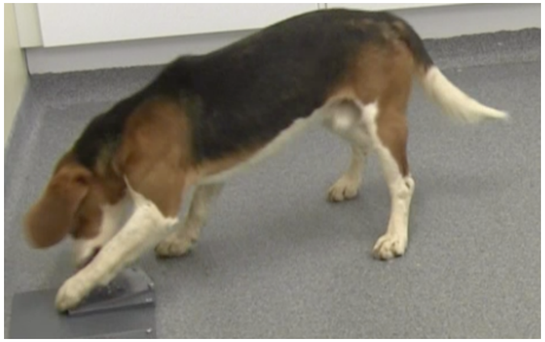

2

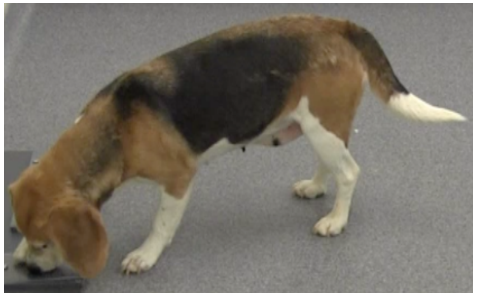

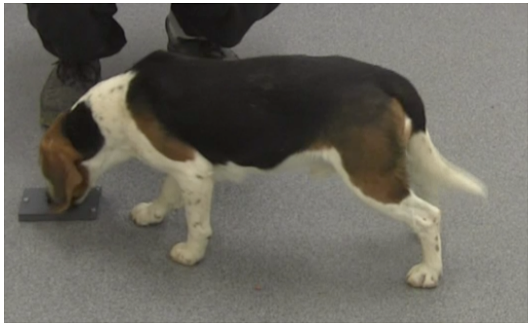

3

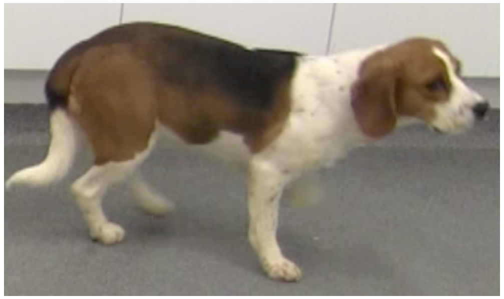

Legs

Score	Name	Description
1	Half covered	At least 3 legs <60% white
2	Mostly covered	At least 3 legs >60%<100% white
3	Completely white	At least 3 legs 100% white and the white extends from the leg on to the body from at least one leg

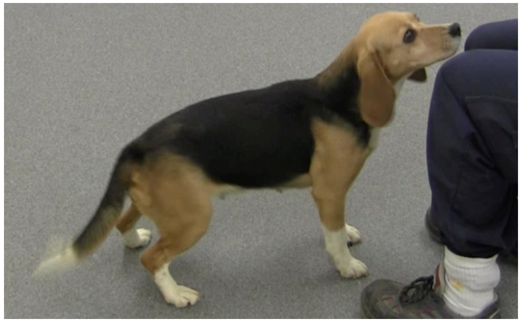

1

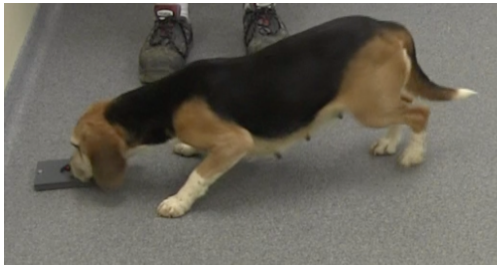

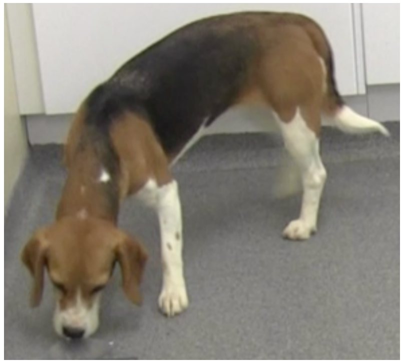

2

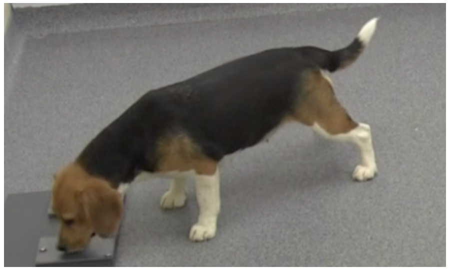

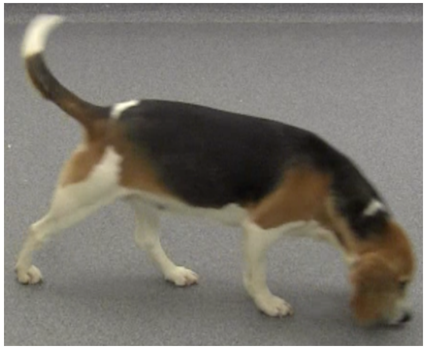

3

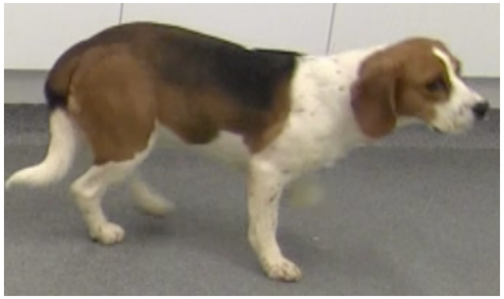

Body

Score	Name	Description
1	Solid	No no or only residual white patches on the body except for the neck area and the ventral side
2	Some white	Only one or two smaller white patch/patches on the body except for the neck area and the ventral side, usually extending from the hind legs
3	White patches	Large white patches, usually on both sides of the body

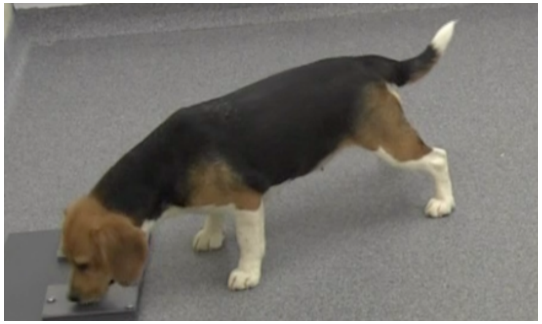

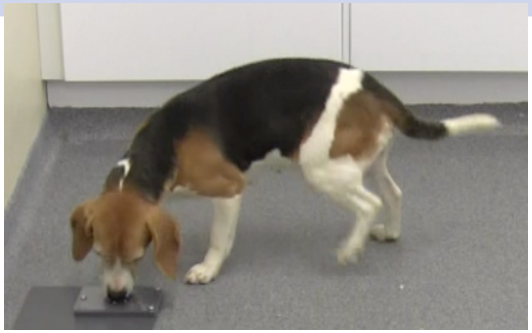

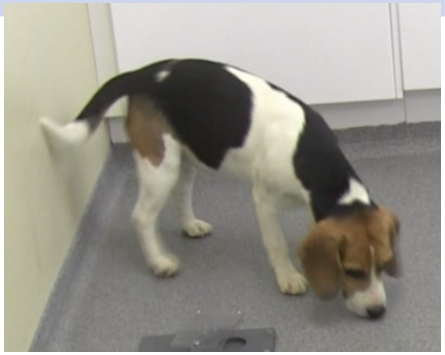

1

2

3

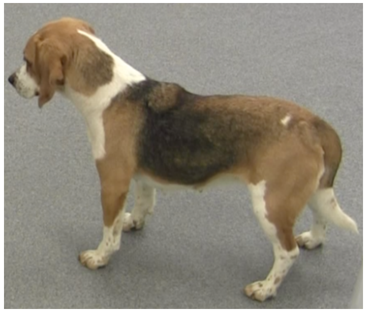

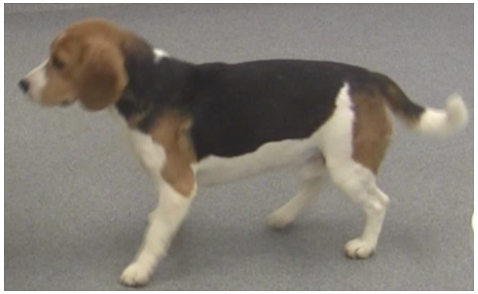

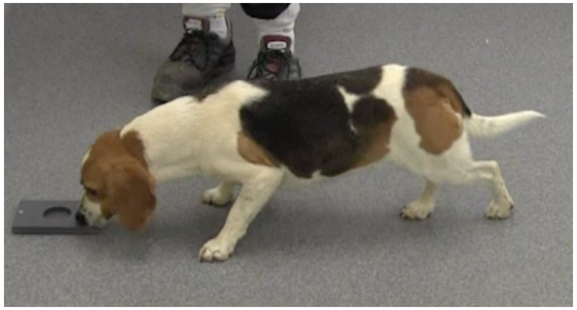

Saddle

Score	Name	Description
1	Solid	Solid black saddle from the neck to the tail base
2	Semi-solid	Mostly solid black saddle from the neck to the tail base, but brown patches occurs
3	Faded	No or mostly no solid black saddle

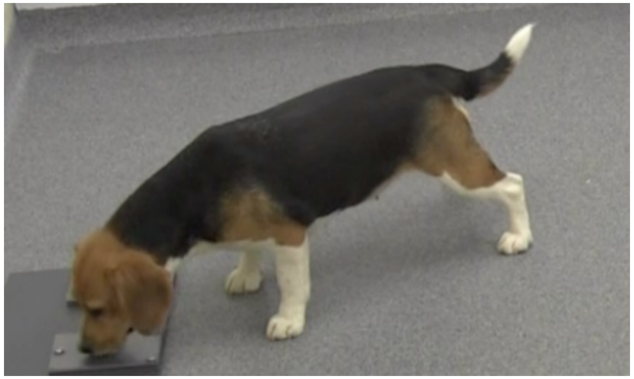

1

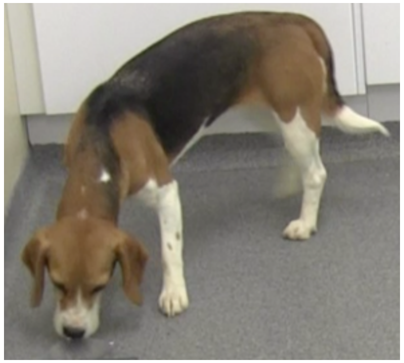

2

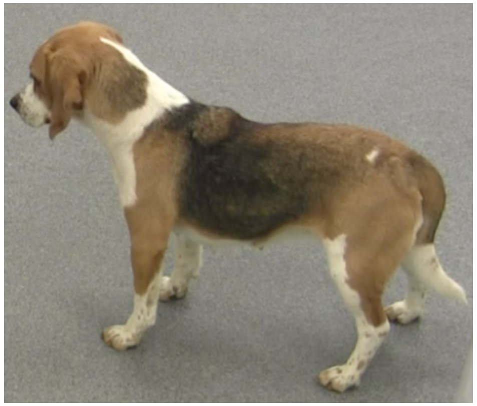

3

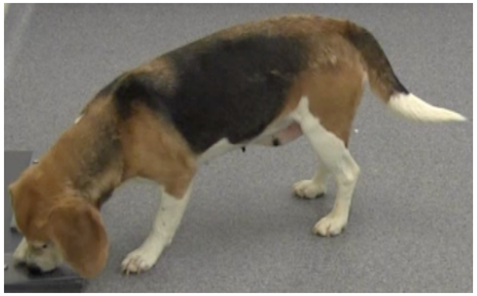

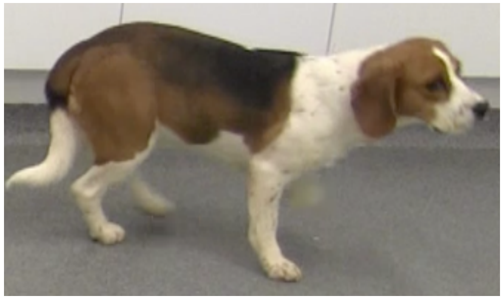

Supplement: Supplementary file 1 [file genes-12-00316-s001.zip › Zip suppl/Phenotype pictures.pdf]
